# Disseminated *Klebsiella pneumoniae* Infection Mimicking Multiple Colonic Subepithelial Lesions: A Case Report

**DOI:** 10.1002/deo2.70431

**Published:** 2026-09-13

**Authors:** Yoshiaki Kobayashi, Tadanobu Nagaya, Mai Iwaya, Hiroshi Ikeuchi, Takuma Okamura, Takeshi Uehara, Yugo Iwaya

**Affiliations:** ^1^ Department of Medicine Division of Gastroenterology Shinshu University School of Medicine Nagano Japan; ^2^ Department of Laboratory Medicine Shinshu University School of Medicine Nagano Japan

**Keywords:** colon, disseminated infection, hypervirulent *Klebsiella pneumoniae*, *Klebsiella pneumoniae*, subepithelial lesion

## Abstract

We report a rare case of disseminated *Klebsiella pneumoniae* infection mimicking multiple subepithelial lesion (SEL)‐like elevations throughout the colon. A 73‐year‐old man was referred for the treatment of diarrhea, edema, and anemia. Colonoscopy revealed numerous SEL‐like elevations from the cecum to the rectum, raising suspicion for malignant or infiltrative disease. Endoscopic ultrasonography demonstrated thickening centered in the deep mucosa and muscularis mucosae, with possible extension into the submucosa. Histological examination showed inflammatory cell infiltration, and bacterial culture of biopsy material identified *K. pneumoniae*. Further systemic evaluation revealed pacemaker lead infection, infective endocarditis, and pyogenic spondylitis, indicating disseminated infection. The colonic lesions improved after targeted antimicrobial therapy and device management. This rare case underscores the importance of considering disseminated bacterial infection in diffuse SEL‐like colonic elevations, particularly when accompanied by systemic infectious manifestations.

## 1 Introduction

Gastrointestinal subepithelial lesions (SELs) characteristically present as intramural growths or elevations covered with intact mucosa [1]. Most cases of infectious enterocolitis exhibit mucosal erythema, edema, erosions, and ulcerations [2], with SEL‐like findings considered rare.

*Klebsiella pneumoniae* is an important opportunistic pathogen associated with a broad range of community‐acquired and healthcare‐associated infections [3]. Although gastrointestinal manifestations of *K. pneumoniae* are uncommon [4], hypervirulent *K. pneumoniae* (hvKp) strains have emerged with enhanced pathogenicity, causing disseminated infection, including liver abscess, endophthalmitis, and meningitis [5].

We herein describe the rare occurrence of disseminated *K. pneumoniae* infection presenting as diffuse colonic SEL‐like elevations and initially mimicking malignant or infiltrative disease.

### 1.1 Case Report

The patient was a 73‐year‐old man with a history of thymoma treated by total thymectomy at 50 years old and resection of recurrent pleural lesions at 59 years old. He had no known history of immunodeficiency. A pacemaker had been implanted at the age of 68 years for complete atrioventricular block.

**FIGURE 1:**
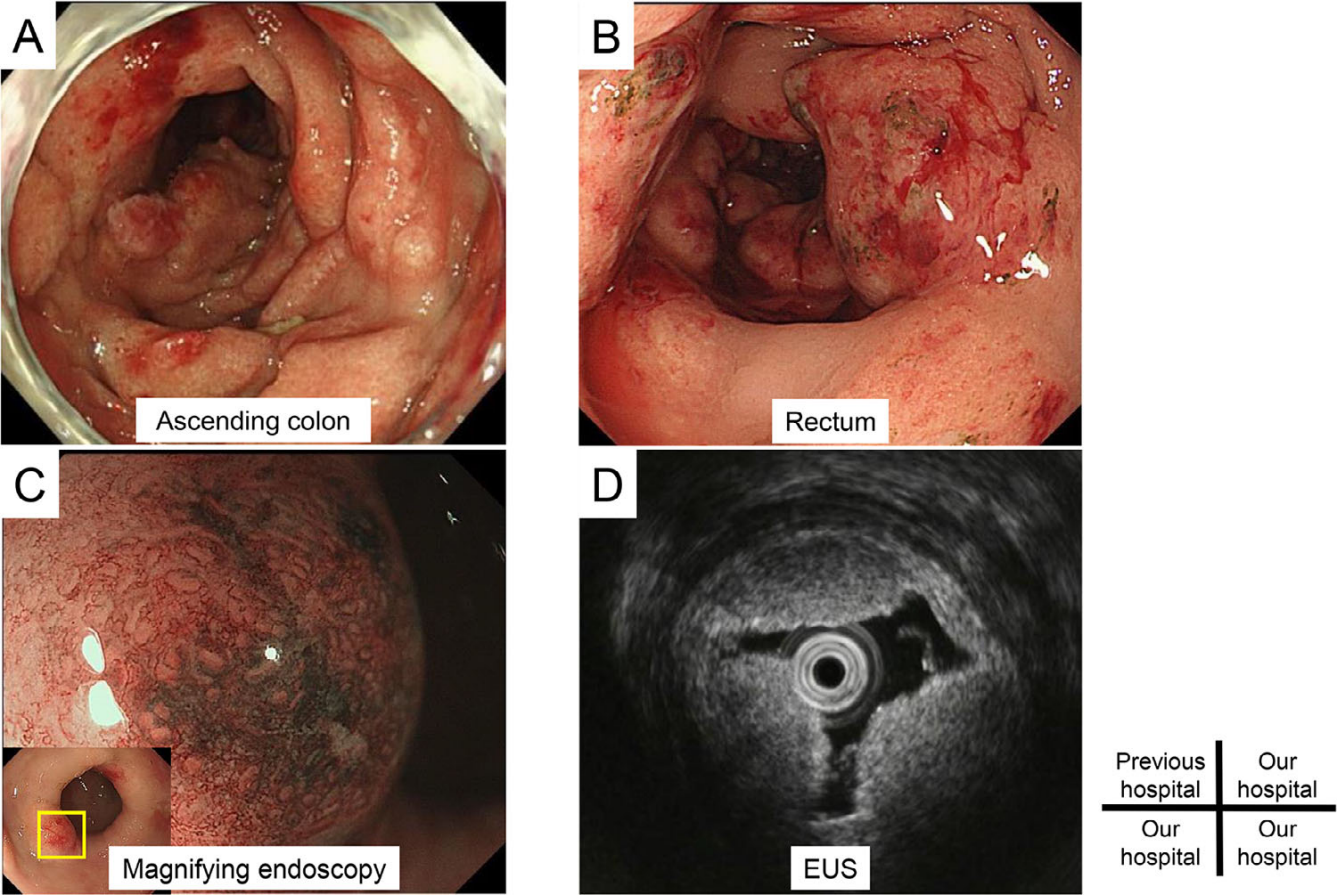
Colonoscopy and endoscopic ultrasonography (EUS) findings: (A) Colonoscopy at the patient's previous hospital revealed multiple subepithelial lesion (SEL)‐like elevations in the colon; (B and C) repeat colonoscopy at our hospital demonstrated progression of the SEL‐like elevations, whereas the mucosal surface architecture remained preserved; and (D) EUS depicted thickening centered in the second layer.

Four months before admission, he experienced persistent diarrhea and progressive edema. Two months prior to admission, he was hospitalized at another medical center for anemia and hypoalbuminemia. Colonoscopy revealed multiple SEL‐like elevations from the cecum to the rectum (Figure 1A). However, a definitive diagnosis was not possible despite extensive examination. He was temporarily discharged after stabilization and then readmitted for worsening general condition 14 days before transfer to our hospital. Blood culture identified *K. pneumoniae*, which prompted ceftriaxone treatment.

**FIGURE 2:**
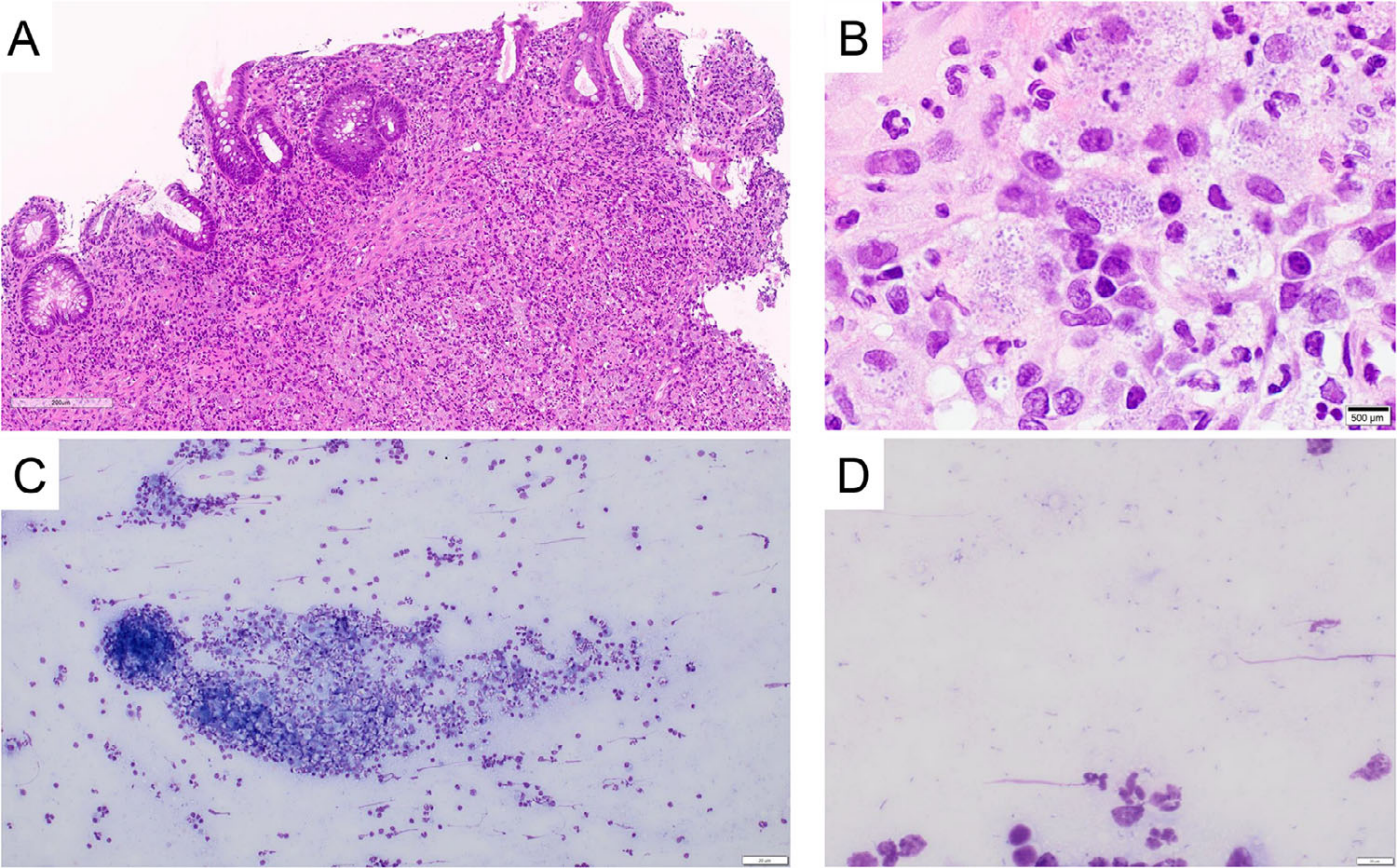
Histopathological findings of colonic mucosal biopsy: (A and B) Histological examination revealed inflamed colonic mucosa with the presence of granular material within histiocytes (hematoxylin and eosin staining); (C and D) Giemsa‐stained imprint smear prepared from a cervical lymph node biopsy specimen revealed numerous rod‐shaped bacteria.

**FIGURE 3:**
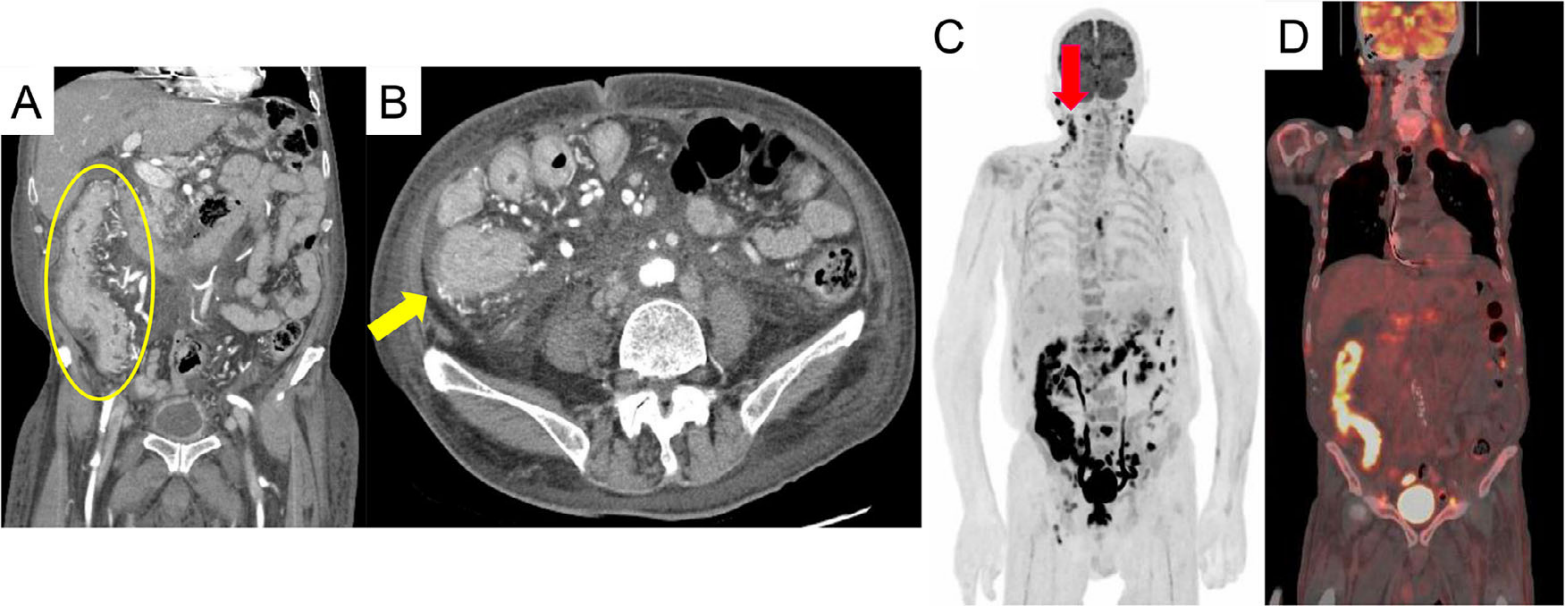
Contrast‐enhanced computed tomography (CT) and positron emission tomography–CT (PET–CT) findings: (A and B) Contrast‐enhanced CT showed a wall thickening of the ascending colon with pericolonic fat stranding (yellow circle, yellow arrow); (C and D) PET–CT demonstrated hypermetabolic activity in systemic lymph nodes, including the cervical lymph nodes (red arrow), and in the ascending colon.

**FIGURE 4:**
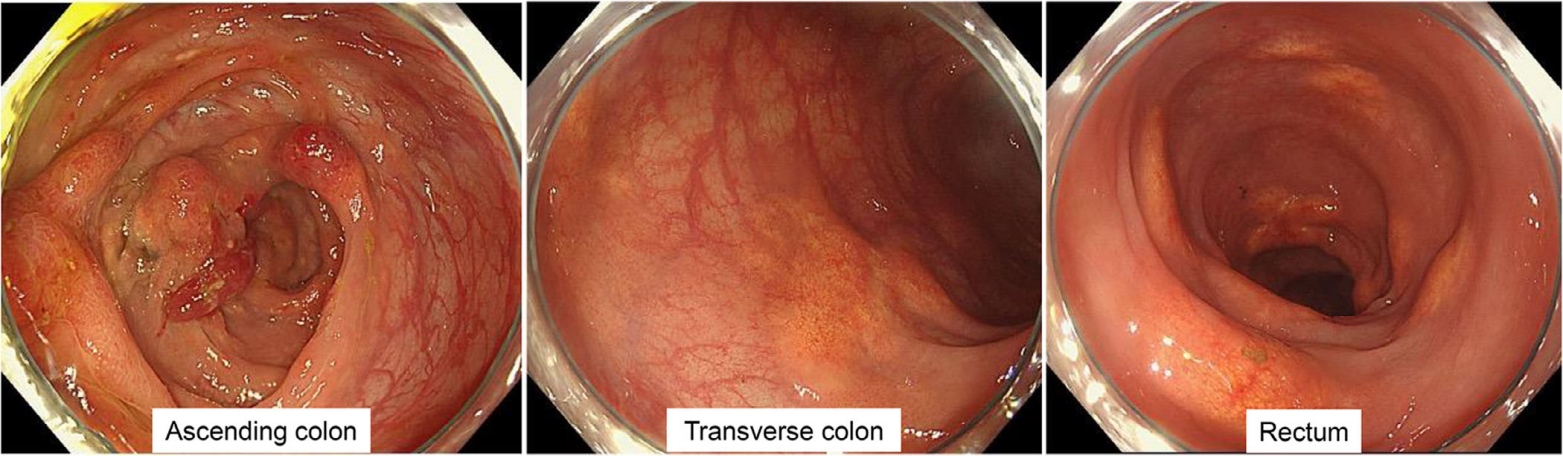
Follow‐up colonoscopy findings. Follow‐up colonoscopy on hospital Day 31 showed an improvement of the multiple SEL‐like elevations.

Upon admission to our department for further evaluation (hospital Day 1), he was afebrile with stable vital signs, bilateral lower extremity edema, and no abdominal tenderness. Laboratory tests revealed anemia, severe hypoalbuminemia, and elevated inflammatory markers (Table 1). Repeat colonoscopy demonstrated the progression of the SEL‐like elevations, with preserved mucosal structure (Figure 1B,C). The masses were firm and exhibited limited movement. Endoscopic ultrasonography demonstrated thickening centered in the second layer, corresponding to the deep mucosa and muscularis mucosae, with possible extension toward the third layer (submucosa) (Figure 1D). Histologically, the colonic mucosa was inflamed and contained numerous histiocytes with fine granular cytoplasmic material (Figure 2A,B). Although neutrophil‐rich granulation tissue was observed, no definite microabscesses or abscess‐like lesions were identified. Contrast‐enhanced computed tomography (CT) showed colonic wall thickening, and positron emission tomography–CT demonstrated increased uptake in the ascending colon and systemic lymph nodes, including the cervical region (Figure 3). No overt small intestinal lesions were apparent on imaging examination, suggesting that the disease predominantly involved the colon. Cervical lymph node biopsy findings excluded malignant lymphoma. A Giemsa‐stained imprint smear disclosed numerous rod‐shaped bacteria (Figure 2C,D). Although no specimen was submitted for microbiological culture, *K. pneumoniae* was subsequently isolated from residual tissue on a sterile dish used for G‐banding (cytogenetic) analysis. Additional systemic evaluation, including transthoracic echocardiography and repeat CT, revealed pacemaker lead infection with infective endocarditis and pyogenic spondylitis. *K. pneumoniae* was isolated from blood cultures, colonic biopsy specimens, and vegetation on the extracted pacemaker lead. Antibiotic therapy was intensified with meropenem and vancomycin, and the infected lead was replaced with an epicardial lead. Follow‐up colonoscopy on hospital Day 31 showed an improvement of the SEL‐like elevations (Figure 4). Concurrent esophagogastroduodenoscopy demonstrated no notable findings, although food residue partially obscured the field of view. Serum albumin levels had also improved from 1.7 g/dL on admission to 2.5 g/dL on hospital Day 31. During this period, he continued treatment primarily for the infective endocarditis. After antimicrobial de‐escalation, the patient completed 3 months of antibiotic therapy for endocarditis and pacemaker lead infection, followed by continuous treatment with minocycline and clindamycin for pyogenic spondylitis. He was discharged on hospital Day 126 in stable condition, with minocycline discontinued 2 months post‐discharge.

**TABLE 1:** Laboratory data at transfer to our hospital (hospital Day 1).

Hematology		Biochemistry		Serology	
**WBC**	**10,090**/µL	**TP**	**6.2** g/dL	**CRP**	**9.29** mg/dL
Neu	85.8%	**Alb**	**1.7** g/dL	Procalcitonin	0.11 ng/mL
Lym	**7.1**%	**AST**	**12** U/L	CEA	1.6 ng/mL
**RBC**	**2.59 × 10^6^**/µL	**ALT**	**8** U/L	**CA19‐9**	**44.3 **U/mL
**Hb**	**7.0** g/dL	ALP^a^	102 U/L	**IgG**	**3342 **mg/dL
**Plt**	**7.1 × 10^4^**/µL	γ‐GTP	17 U/L	IgA	170 mg/dL
ESR		T‐Bil	0.45 mg/dL	IgM	56 mg/dL
**ESR (1 h)**	**24 **mm	**AMY**	**25** U/L	HIV Ag/Ab	(—)
Coagulation		LDH	156 U/L	**sIL‐2R**	**1192** U/mL
**PT**	**66.8**%	**CHE**	**34** U/L	Antinuclear antibody	(—)
**APTT**	**48.3 **s	**CK**	**9** U/L	RF	(—)
FIBG	214 mg/dL	**T‐CHO**	**41** mg/dL	MMP‐3	(—)
**D‐dimer**	**2.9** µg/mL	**BUN**	**32.1** mg/dL	PR3‐ANCA	(—)
		Cre	0.78 mg/dL	MPO‐ANCA	(—)
		Na	136 mEq/L	ACE	5.1 U/L
		K	3.6 mEq/L		
		**Ca**	**7.0** mg/dL		
		**Fe**	**17** µg/dL		

*Note*: Bold values indicate abnormal results

Abbreviations: γ‐GTP, gamma‐glutamyl transferase; ACE, angiotensin converting enzyme; Alb, albumin; ALP, alkaline phosphatase; ALT, alanine aminotransferase; AMY, amylase; APTT, activated partial thromboplastin time; AST, aspartate aminotransferase; BUN, blood urea nitrogen; Ca, calcium; CA19‐9, carbohydrate antigen 19‐9; CEA, carcinoembryonic antigen; CHE, cholinesterase; CK, creatine kinase; Cre, creatinine; CRP, C‐reactive protein; ESR, erythrocyte sedimentation rate; Fe, iron; FIBG, fibrinogen; Hb, hemoglobin; HIV Ag/Ab, human immunodeficiency virus antigen/antibody; IgA, immunoglobulin A; IgG, immunoglobulin G; IgM, immunoglobulin M; K, potassium; LDH, lactate dehydrogenase; Lym, lymphocytes; MMP‐3, matrix metalloproteinase‐3; MPO‐ANCA, myeloperoxidase‐anti‐neutrophil cytoplasmic antibody; Na, sodium; Neu, neutrophils; Plt, platelets; PR3‐ANCA, proteinase 3‐anti‐neutrophil cytoplasmic antibody; PT, prothrombin time; RBC, red blood cells; RF, rheumatoid factor; sIL‐2R, soluble interleukin‐2 receptor; T‐Bil, total bilirubin; T‐CHO, total cholesterol; TP, total protein; WBC, white blood cells.

^a^Measured using the International Federation of Clinical Chemistry method.

## 2 Discussion

To our knowledge, this is the first reported account of disseminated *K. pneumoniae* infection presenting with multiple colonic SEL‐like elevations, accompanied by pacemaker lead infection, infective endocarditis, and pyogenic spondylitis. The clinical significance of this case lies not only in its rarity but also in its potential to mimic malignant or infiltrative disorders of the colon, thereby creating a substantial diagnostic challenge. In the present patient, the initial endoscopic impression raised concern for diseases, such as malignant lymphoma, whereas the final diagnosis depended on the recognition of systemic infection and microbiological identification of *K. pneumoniae* from biopsy‐derived material.

*K. pneumoniae* is known to cause a wide spectrum of infections, ranging from pneumonia and urinary tract infections to liver abscesses [6]. In particular, hvKp exhibits enhanced virulence and a propensity for metastatic spread to distant sites that may lead to liver abscesses, endophthalmitis, and systemic infection involving multiple organs [5]. Infections can occasionally cause SEL‐like lesions in the colon [7], although no reports to date have described *K. pneumoniae* infection presenting as SEL‐like elevations. In the present case, the discovery of pacemaker lead infection, infective endocarditis, and pyogenic spondylitis suggested sustained bacteremia [8, 9]. The precise route of infection remains uncertain; however, two potential mechanisms may explain the colonic involvement. One possibility is that prolonged bacteremia resulted in hematogenous dissemination with bacterial seeding in the colonic submucosa, ensuing submucosal inflammation, and the ultimate formation of diffuse SEL‐like elevations throughout the colon. Alternatively, extensive colonic inflammation might have facilitated bacterial translocation, with the colon serving as the portal of entry or primary intestinal focus and subsequent systemic dissemination. The diffuse distribution of SEL‐like lesions throughout the colon may support hematogenous dissemination as a possible mechanism. We were unable to determine the precise route of infection in this case. Notably, the patient remained afebrile despite extensive bacterial dissemination, possibly due to older age, severe malnutrition, and prior antimicrobial therapy. Indeed, both advanced age and malnutrition are known to blunt the febrile response [10, 11]. Our diagnosis was supported by bacterial identification in biopsy specimens and marked improvement following antimicrobial therapy. Although liver abscess, endophthalmitis, and other typical manifestations of hvKp were not observed, hvKp might also present with such atypical manifestations as disseminated multi‐organ infection [5]. The present patient exhibited several clinical features suggestive of hvKp, including disseminated multi‐organ infection and severe disease without apparent immunodeficiency, which supported the possibility of hvKp involvement. However, other typical manifestations were absent, and microbiological confirmation was unavailable.

The string test is considered a useful, rapid, and simple screening method for hypervirulent strains; however, as we did not suspect hvKp at the time of biopsy, no test was performed during the acute phase. It should be noted that *K. pneumoniae* may show a negative string test result while displaying a high virulence phenotype, and vice versa [12]. Ideally, genotypic diagnosis using polymerase chain reaction assays or the quantitative measurement of siderophore production is included as well [5].

It is conceivable that host factors may also have contributed to the development and persistence of the disseminated infection. Severe malnutrition could have impaired host defense mechanisms. As lymphocyte subset analysis and other detailed immunological evaluation were unavailable, subtle immune dysfunction could not be completely excluded. Malakoplakia was also considered in the differential diagnosis due to the presence of numerous histiocytes in the biopsy specimens. However, no Michaelis–Gutmann bodies were identified, and pathological findings were insufficient for a definitive diagnosis.

We searched PubMed/MEDLINE using the terms “bacterial infection,” “colon,” and “subepithelial lesion” and identified reports of localized SEL‐like elevations from infections, such as tuberculosis [13, 14], with no cases of diffuse involvement of the entire colon. This highlights both the novelty of our case and the importance of considering *K. pneumoniae* infection for similar presentations.

In summary, disseminated bacterial infection, including *K. pneumoniae*, should be considered in the differential diagnosis of diffuse colonic SEL‐like elevations, especially when accompanied by bacteremia or other systemic infectious manifestations. Careful systemic evaluation and microbiological examination of biopsy‐derived material may be crucial for establishing the diagnosis and guiding appropriate treatment.

## Author Contributions

**Yoshiaki Kobayashi**: patient care, manuscript writing. **Tadanobu Nagaya**: manuscript writing, manuscript editing, manuscript submission. **Mai Iwaya**: pathological evaluation. **Hiroshi Ikeuchi**: patient care, manuscript review. **Takuma Okamura**: patient care, manuscript review. **Takeshi Uehara**: pathological evaluation. **Yugo Iwaya**: manuscript review, manuscript editing, supervision.

## Funding

The authors have nothing to report.

## Ethics Statement

The authors have nothing to report.

## Consent

Written informed consent was obtained from the patient for the publication of this case report and accompanying images. A copy of the written consent form is available for review by the Editor‐in‐Chief of this journal upon request.

## Conflicts of Interest

The authors declare no conflicts of interest.

## Data Availability

The data that support the findings of this study are available on request from the corresponding author. The data are not publicly available due to privacy or ethical restrictions.
